# Sleep Quality Assessment in Trauma Patients Requiring Intensive Care: A Prospective Observational Study

**DOI:** 10.7759/cureus.111889

**Published:** 2026-07-01

**Authors:** Sathiya G Priya, Yudhyavir Singh, Kapil Dev Soni, Richa Aggarwal, Chhavi Sawhney, Babita Gupta

**Affiliations:** 1 Anesthesiology and Critical Care, All India Institute of Medical Sciences, New Delhi, New Delhi, IND; 2 Division of Trauma Anesthesiology and Critical Care, All India Institute of Medical Sciences, New Delhi, New Delhi, IND; 3 Critical and Intensive Care, All India Institute of Medical Sciences, New Delhi, New Delhi, IND

**Keywords:** delirium, icu, noise, pain, rcsq, sleep quality, sleep questionnaire, trauma

## Abstract

Background

Sleep is vital for healing, cognitive function, immunity regulation, hormonal balance, and waste clearance. Sleep deprivation is common among intensive care unit (ICU) patients, particularly those receiving mechanical ventilation. Fragmented sleep, pain, noise, and other factors adversely affect recovery and outcomes. Trauma patients often experience increased pain from their injuries. As most sleep studies have been conducted in general and surgical ICUs, but not in trauma patients, a sleep quality assessment study was planned for trauma patients requiring intensive care. The primary objective was to evaluate sleep quality in trauma patients admitted to the ICU. The secondary objectives were to assess sleep quality changes during ICU stay, identify factors affecting sleep, measure environmental noise levels, and determine the incidence of delirium.

Methodology

A single-center, prospective observational study was conducted at a level I trauma ICU of a tertiary care hospital involving 80 trauma patients from July 2022 to June 2024. Patients were assessed using the Richmond Agitation and Sedation Scale (RASS) to evaluate sedation and agitation levels. Those with a RASS score of -1 to +1 were screened for delirium using the Confusion Assessment Method (CAM-ICU). Delirium-free patients (CAM-ICU score of 0-2) were recruited, and their sleep quality was assessed using the Richards-Campbell Sleep Questionnaire (RCSQ) over three days. Poor sleep was defined as RCSQ <50, and the poor sleep and good sleep groups were analyzed. Data were expressed as mean ± SD or median (IQR) or percentage as appropriate. Sleep groups were compared using Student’s t-test for normally distributed continuous variables, Mann-Whitney U test for non-parametric variables, and ANOVA test for repeated measures, while categorical data were compared using Fisher’s exact test.

Results

The mean RCSQ score was 49.87± 5.35 on day one, and the overall mean RCSQ score of the study population was 49.57 ± 4.95, indicating generally poor sleep quality among trauma ICU patients. Poor sleep (RCSQ <50) was found in 52.5% of patients on the first day, 45% on the second day, and 43.75% on the third day. The mean RCSQ score was significantly lower in the poor sleep group (45.87 ± 2.60) compared with the good sleep group (54.29 ± 3.90) (p = 0.001). Overall, the mean age was 41.42 ± 17.04 years and was comparable between the groups, with a male predominance (82.5%, 66). Noise was the most common factor (n = 52), followed by pain (n = 37) and light (n = 18), which kept the patients awake. Mean noise level in the ICU over three days was 48.7 ± 2.04 dB, which was above the WHO recommendations. Delirium cumulative incidence was 20%, and overall mortality was 11.25%. This was not significant between the poor and good sleep groups, though higher mortality was reported in the poor sleep group.

Conclusions

Trauma patients requiring ICU care reported poor sleep quality, with only minimal improvement during their ICU stay. Noise was identified as the most significant sleep disruptor, followed by light exposure and pain. Noise levels in the ICU exceeded the recommended limits, with significantly higher noise levels reported during the daytime compared with nighttime. Sleep quality did not significantly influence mortality in trauma patients. Better sleep management is required with targeted interventions, though larger trials are needed to confirm its impact on clinical outcomes.

## Introduction

Sleep plays an essential role in physical restoration, cognitive function, immune regulation, and overall well-being. Sleep helps remove metabolic waste, peaks growth hormone for healing, and increases cortisol secretion [1,2]. In healthy individuals, sleep typically involves four to six cycles, each lasting 90-100 minutes, alternating between non-rapid eye movement (NREM) and rapid eye movement (REM) phases, resulting in a total sleep duration of seven to eight hours per night. Rapid eye movement sleep, which makes up about 20% of total sleep and mainly happens in the morning, is believed to play a key role in memory restructuring [3].

Numerous methods have been used to assess the quality of sleep in the intensive care unit (ICU). Polysomnography remains the gold standard for sleep quality assessment; however, in the ICU scenario, it is cumbersome and difficult to use [4]. Other methods use questionnaires as a tool. The Richards-Campbell Sleep Questionnaire (RCSQ) is the most commonly used tool for evaluating ICU patients’ own perception of sleep [4]. The score is a six-item self-report questionnaire designed to assess perceived sleep depth, sleep latency, frequency of awakenings, sleep efficiency, sleep quality, and noise exposure. Each RCSQ item is evaluated using a visual analog scale ranging from 0 to 100 mm, where greater values indicate improved sleep quality. The average score derived from the six items is referred to as the total RCSQ score, which serves as an indicator of overall sleep quality. The original RCSQ was validated for reliability and validity against polysomnographic recordings in 70 critically ill patients [5]. Multiple studies have confirmed RCSQ as the most clinically useful tool for sleep observation in the ICU, even in intubated patients [6-9].

The Sleep in the ICU Questionnaire (SICUQ) is another tool to evaluate sleep quality in critically ill patients and collect data on factors affecting sleep in the ICU, including environmental factors and routine patient care activities [10]. Patients rate the overall quality of their sleep at home and in the ICU on a scale from 1 to 10, with 1 representing “poor” and 10 indicating “excellent.” Validation of the SICUQ against polysomnography and through reproducibility studies has not yet been done.

Adequate sleep is essential for hospitalized patients, particularly patients who require ICU care. However, sleep in such patients is frequently disrupted and often overlooked and inadequately addressed. Critically ill patients lack deep restorative REM sleep [11,12]. The prevalence of sleep disorders is more than 50% in critically sick patients and septic patients admitted in the ICU [10,13]. About 38.5% of patients who survived critical illness and required mechanical ventilation for at least 48 hours reported poor sleep quality, 40% of these patients reported frequent nighttime awakenings, and 35% reported difficulty falling asleep during their ICU stay [1]. Sleep deprivation results in the release of inflammatory cytokines, which result in adverse cardiovascular outcomes and impaired immunological responses [14]. Multiple factors contribute to poor sleep in the ICU setting, including delirium from organic causes, underlying medical conditions, environmental noise, unfamiliar surroundings, mechanical ventilation, sedative use, and the absence of familiar individuals [5,15,16].

There is a paucity of research addressing sleep quality in trauma patients admitted to intensive care. Therefore, this study was planned to assess sleep quality among trauma patients admitted to the ICU. The primary objective of our study was to assess the quality of sleep in trauma patients admitted to the ICU using RCSQ as the tool. Our secondary objectives were to assess the changes in the sleep quality, noise level, incidence of delirium, and factors affecting sleep quality in trauma patients.

## Materials and methods

This prospective, single-center observational study was conducted at a level 1 trauma center following approval from the Institutional Ethics Committee (approval number: IECPG-449/30.06.2022) and registration with the Clinical Trials Registry-India (CTRI/2022/08/045023), spanning July 2022 to June 2024. Informed and written consent was obtained from the patients, their relatives, or legal guardians. We included all adults aged 18 or older who sustained trauma and were admitted to the trauma ICU with an expected length of stay in the ICU of greater than 24 hours. Patients with a history of chronic neurological illness, drug abuse, sleep disorder, chronic kidney disease, dementia, intubated head injury, non-intubated patients with a Glasgow Coma Scale (GCS) score <15 or <12 in tracheostomized, or those refusing to provide consent were excluded from the study.

Eligible patients were assessed using the Richmond Agitation and Sedation Scale (RASS) to quantify sedation and agitation (used with permission from the original developers) [17]. Sedation was practiced as per the hospital ICU policy, and it was decreased/minimized in the morning hours from 7 am to 10 am to achieve a RASS of -1 to +1. Patients who had a RASS score of -1 to +1 were screened using the Confusion Assessment Method for ICU (CAM-ICU) for the presence of delirium [18]. If CAM-ICU (free to use for academic research and non-commercial purposes) was positive, those patients were excluded from the study. If CAM-ICU was negative, then the patient’s sleep quality was assessed using the RCSQ (used with permission from the original developers) [5]. RCSQ assessment was performed every morning between 7 am and 10 am from day one of the patient’s recruitment to day three of their ICU stay. Hindi translation of the RCSQ tool was performed and verified with two Hindi translators. RCSQ score comprises six parameters that include sleep depth, latency, awakenings, returning to sleep, quality of sleep, and noise. A maximum score of 100 was given to each domain.

Delirium was assessed consecutively once daily for three days using the CAM-ICU. Pain was assessed using the Numeric Pain Rating Scale (NPRS) for three days between 7 am and 10 am daily [19]. Acute Physiology and Chronic Health Evaluation II ( APACHE-II), Sequential Organ Failure Assessment (SOFA), and Injury Severity Score (ISS) were calculated and noted after ICU admission [20-22]. The noise level in the ICU was assessed at the bedside of patients twice daily over three consecutive days using the National Institute for Occupational Safety and Health, Sound Level Meter (NIOSH SLM) mobile application on an iPhone 14. Measurements were conducted at 10:00 pm and 7:00 am, corresponding with periods when sedation was reduced for RCSQ evaluation. NIOSH SLM has been used in iOS-based phones and found to be most effective in measuring sound levels [23,24]. Patients were followed up until the point of discharge from the ICU or day three after enrolment in the study, whichever was the earliest.

Sleep quality definition

Multiple studies have defined the quality of sleep using the RCSQ score. Naik et al. classified patients with an RCSQ score <50 as having poor sleep [25]. Similarly, another study defined good sleep as an RCSQ score ≥50, reporting a sensitivity of 88.24%, a specificity of 86.67%, and an area under the receiver operating characteristic (ROC) curve of 0.92 (95% confidence interval) [5]. Based on this, patients were classified into good and poor sleep groups. Their demographic, clinical characteristics, and clinical outcome parameters were compared between the two groups.

Data collection

Demographic data such as age, gender, ICU stays, and length of hospital stays were noted. Baseline ICU variables such as SOFA, APACHE II, GCS, RASS, ISS Scores of the patients were calculated and noted on day one of their ICU stay. Patients requiring mechanical ventilation and those who had undergone tracheostomy were also included. Pain score (using NPRS), along with sedation and analgesia used and their dosages used, were captured. Mortality at 28 days was also noted.

Sample size

The sample size calculation was based on a previously published mean RCSQ score of 51.6 ± 13.5 in an ICU population [25]. To estimate the mean RCSQ score with 95% confidence and an absolute precision (margin of error) of ±3 points, a minimum sample size of 78 patients was found to be sufficient. Consequently, the study enrolled 80 eligible patients to account for potential attrition and ensure sufficient statistical power. This methodology enabled accurate assessment of sleep quality in the trauma ICU cohort, maintaining accuracy and precision within clinically relevant parameters.

Statistical analysis

All statistical analyses were conducted using SPSS software, version 23.0 (IBM Corp., Armonk, NY, USA). Continuous data was expressed as mean ± standard deviation (SD) when normally distributed and compared between the groups using the independent t-test. For continuous data that did not follow a normal distribution, results are presented as the median with interquartile range (interquartile range), and group comparisons were conducted using the Mann-Whitney U test and analysis of variance test as appropriate. For paired continuous data with a normal distribution, a paired t-test was applied. Categorical variables were expressed as frequencies and percentages and were compared using Fisher’s exact test. Variables with a p-value <0.05 were considered statistically significant.

## Results

**Figure 1:**
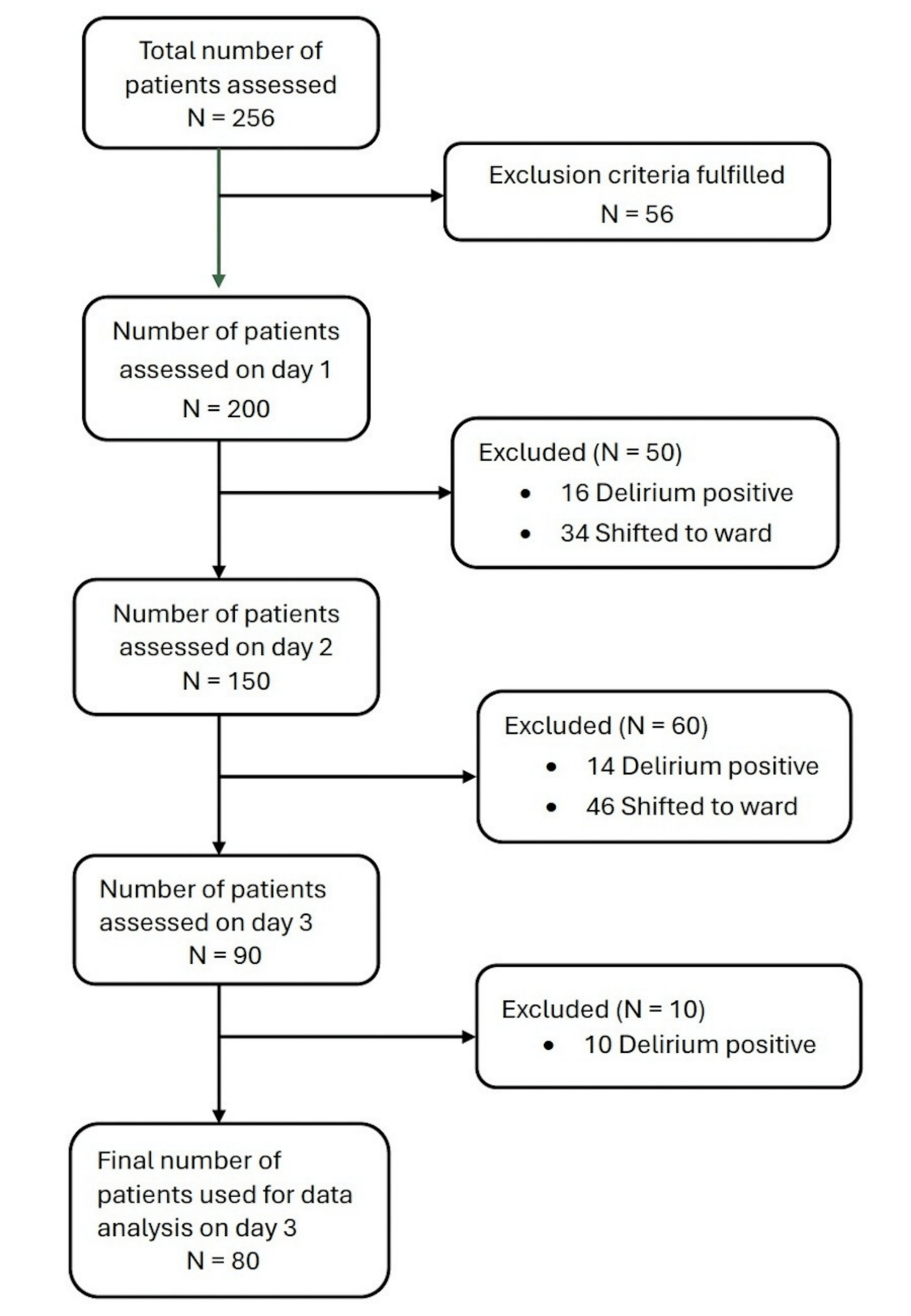
Flowchart illustrating study recruitment.

A total of 80 trauma patients who fulfilled all criteria were enrolled in this study, as illustrated in Figure 1.

The mean age of the study participants was 41.42 years (SD = 17.04), and the majority of patients were male (82.5%, n = 66). Based on the mean RCSQ score, patients were categorized into poor sleep (RCSQ <50) and good sleep (RCSQ >50) groups. Overall, 42 (52.5%) patients had poor sleep quality, while 38 (47.5%) patients had relatively better sleep quality during their ICU stay (Table 1).

**Table 1:** Demographic and clinical characteristics of trauma patients according to sleep quality. Poor sleep quality: mean RCSQ score <50; good sleep quality: mean RCSQ score ≥50. Data are expressed as mean ± SD or percentage as appropriate. *: Student’s t-test; ^l^: Fisher’s exact test; ^#^: Mann-Whitney test. A p-value <0.05 is considered significant. APACHE II: Acute Physiology and Chronic Health Evaluation Score II; SOFA: Sequential Organ Failure Assessment score; ISS: Injury Severity Score; RCSQ: Richards-Campbell Sleep Questionnaire

Parameter		Total patients (n = 80)	Poor sleeper (n = 42)	Good sleeper (n = 38)	Test statistics	P-value
Age (years)		41.42 ± 17.04	41.23 ± 17.66	41.63 ± 16.55	t = -0.102	0.54^*^
Gender	Male	66 (82.5%)	35	31	χ² = 0.42	1^l^
	Female	14 (17.5%)	7	7		
APACHE II		3.32 ± 2.79	3.5 ± 2.76	3.13 ± 2.84	Z = 0.812	0.41^#^
SOFA		1.09 ± 1.29	1.04 ± 1.32	1.13 ± 1.27	Z = -0.417	0.67^#^
ISS		23.32 ± 13.59	23.64 ± 13.88	22.97 ± 13.42	z = 0.372	0.71^#^
Mechanical ventilation		24 (30%)	14 (33%)	10 (26%)	x = 0.298	0.72^l^
Length of ICU stay (days)		11.24 ± 7.14	11.69 ± 8.14	10.73 ± 5.90	z = 0.232	0.81^#^
Length of hospital stay (days)		21.67 ± 8.59	21.61 ± 8.48	21.73 ± 8.82	z = 0.048	0.96^#^
Noise level (dB)		48.70 ± 2.04	49.52 ± 2.87	49.06 ± 4.20	z = -0.400	0.68^#^
Pain score		5.87 ± 0.9	5.88 ± 0.83	5.86 ± 0.99	z = 1.24	0.21^#^
Mean RCSQ scores		49.57 ± 4.95	45.87 ± 2.60	54.29 ± 3.90	t = - 11.44	0.001^*^
Mortality (28 days)		9 (11.25%)	7 (16.67 %)	2 (5.26%)	χ² = 2.59	0.103^l^

The demographic and baseline clinical characteristics were comparable between the two groups. The mean age in the poor sleep group was 41.23 ± 17.66 years compared to 41.63 ± 16.55 years in the good sleep group (p = 0.54). Male predominance was observed in both groups without any statistically significant difference. Severity scores, such as APACHE II, SOFA, and ISS, were also similar between groups. The mean APACHE II score was 3.5 ± 2.76 in the poor sleep group and 3.13 ± 2.84 in the good sleep group (p = 0.41). Similarly, SOFA scores and ISS scores did not differ significantly between the groups (p = 0.67 and p = 0.71, respectively).

Mechanical ventilation was required in 24 (30%) patients, of whom 14 belonged to the poor sleep group and 10 to the good sleep group. However, the requirement of mechanical ventilation did not significantly affect sleep quality (p = 0.72). Likewise, ICU length of stay and total hospital stay were comparable between the two groups. Patients with poor sleep had a mean ICU stay of 11.69 ± 8.14 days compared to 10.73 ± 5.90 days in the good sleep group (p = 0.81). Mean hospital stay was also similar between the two groups (21.61 ± 8.48 vs. 21.73 ± 8.82 days; p = 0.96).

The overall mean RCSQ score of the study population was 49.57 ± 4.95, indicating generally poor sleep quality among trauma ICU patients. The mean RCSQ score was significantly lower in the poor sleep group (45.87 ± 2.60) compared with the good sleep group (54.29 ± 3.90) (p = 0.001). Mortality was numerically higher in the poor sleep group (16.67%) compared with the good sleep group (5.26%), although this difference did not reach statistical significance (p = 0.103).

**Figure 2:**
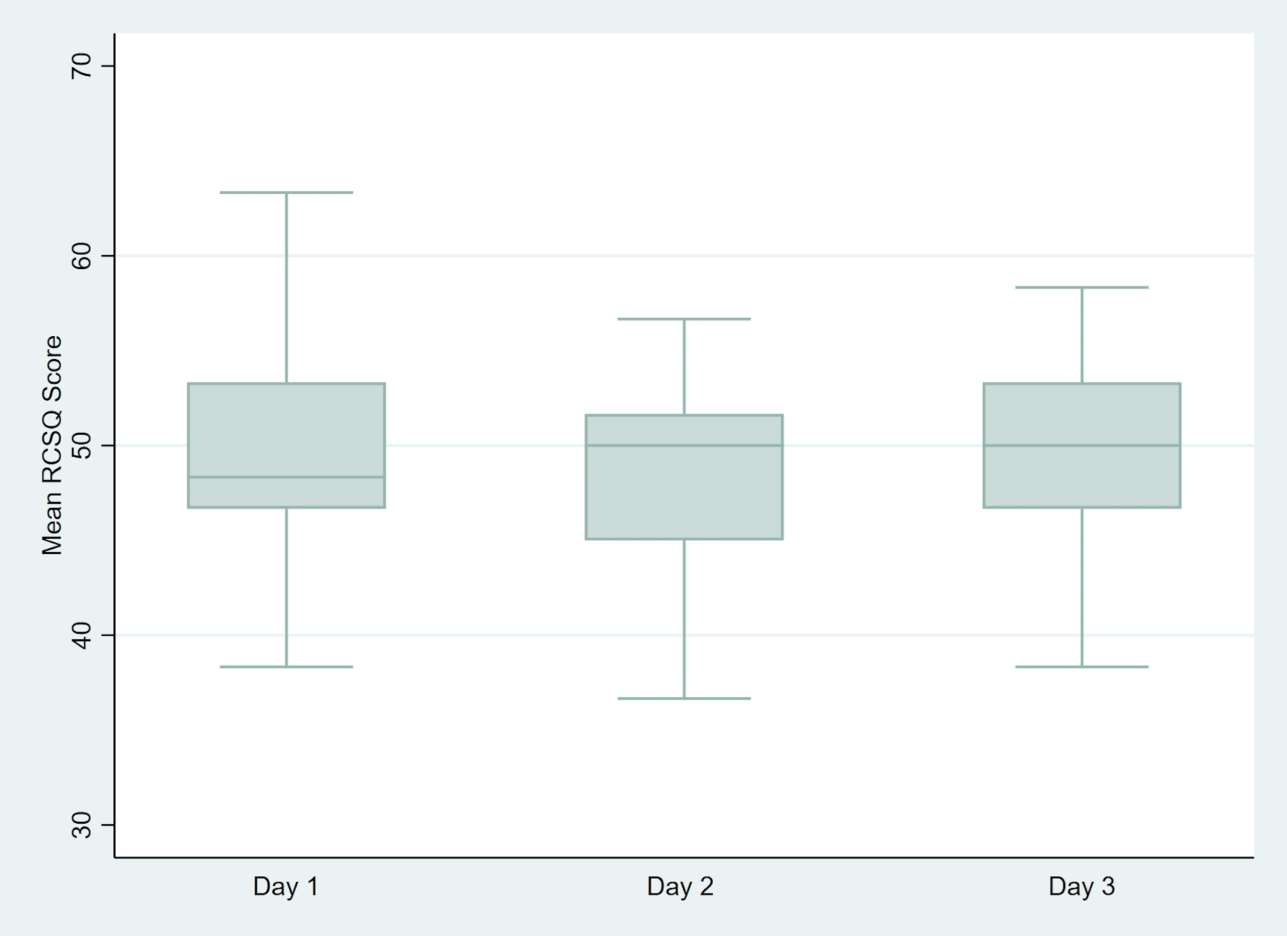
Comparison of mean RCSQ scores across three consecutive ICU days. Box plots depict median values, interquartile ranges, and minimum–maximum values of RCSQ scores on days one, two, and three. Higher RCSQ scores correspond to better perceived sleep quality. RCSQ: Richards-Campbell Sleep Questionnaire; ICU: intensive care unit

Sleep quality assessment over the three days of ICU stay showed persistently poor sleep quality. The mean RCSQ score on day one was 49.87 ± 5.35, which slightly decreased to 48.85 ± 4.79 on day two and marginally improved to 50 ± 4.67 on day three. However, the overall three-day mean RCSQ of 49.57 ± 3.52 indicated generally poor sleep in this trauma cohort. Poor sleep was reported in 52.5% of patients on day one, 45% on day two, and 43.75% on day three, indicating that a substantial proportion of trauma ICU patients continued to experience poor sleep throughout their ICU stay (Table 2, Figure 2).

**Table 2:** Mean RCSQ scores and incidence of poor sleep during the first three days of ICU stays. Data are expressed as mean ± SD or number (%), as appropriate. RCSQ: Richards-Campbell Sleep Questionnaire; ICU: intensive care unit

Day	Mean RCSQ	Poor sleep (RCSQ <50)	Good sleep (RCSQ >50)
Day 1	49.87 ± 5.35	42 (52.5%)	38 (48.5%)
Day 2	48.85 ± 4.79	36 (45%)	44 (55%)
Day 3	50 ± 4.67	35 (43.75%)	45 (47.25%)

**Figure 3:**
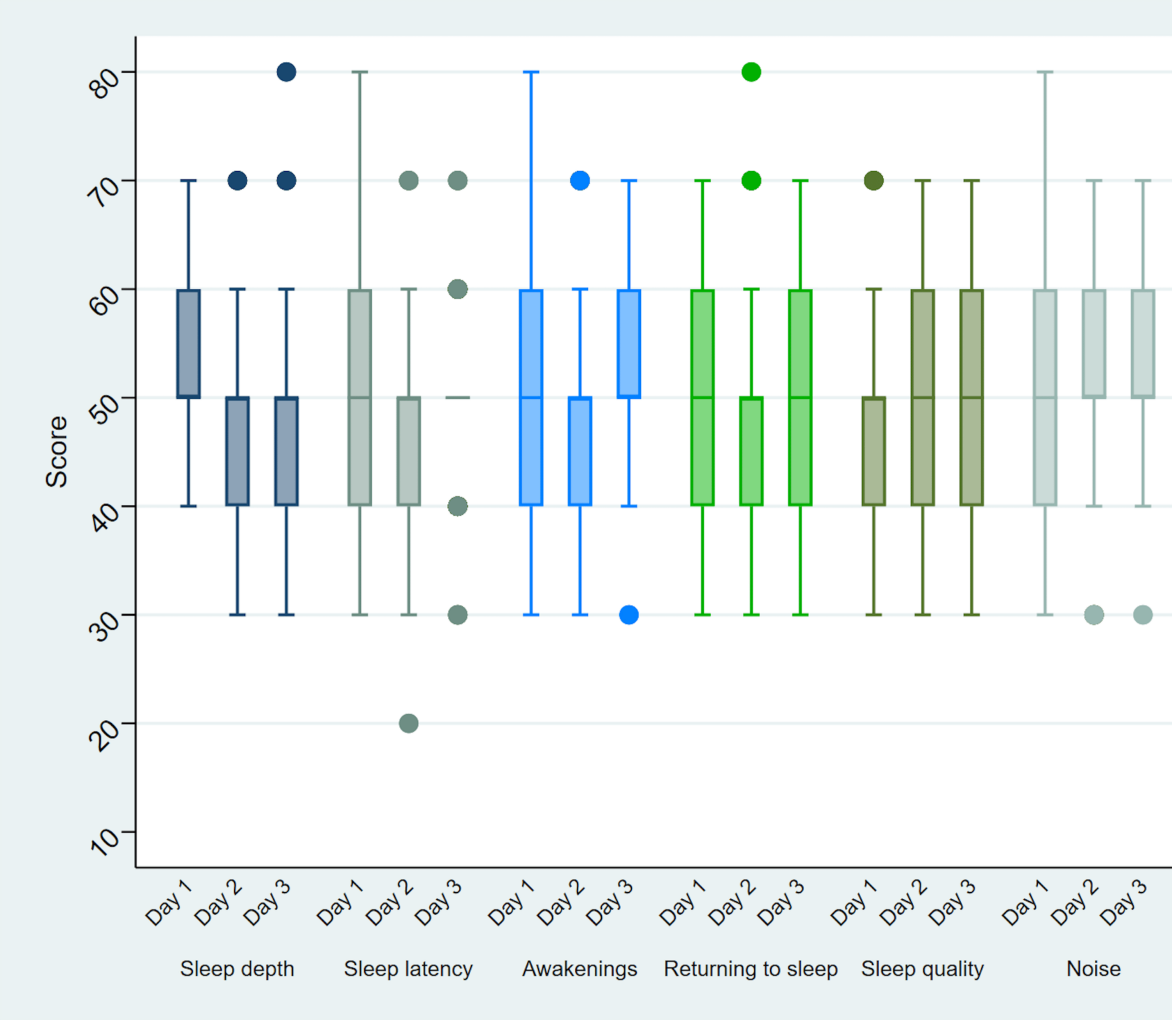
Temporal changes in subjective sleep characteristics over the three days of ICU stay. Box plots show the distribution of six RCSQ domain scores (sleep depth, sleep latency, awakenings, returning to sleep, sleep quality, and noise levels) across days 1–3. The central line represents the median, boxes indicate the interquartile range (25th–75th percentile), whiskers denote the range excluding outliers, and dots represent outlying observations. Higher RCSQ scores reflect better perceived sleep quality. RCSQ: Richards-Campbell Sleep Questionnaire; ICU: intensive care unit

Analysis of individual RCSQ domains over the three days showed no statistically significant differences in sleep depth, sleep latency, awakenings, returning to sleep, perceived sleep quality, or noise perception (Figure 3, Table 3).

**Table 3:** Sleep quality characteristics using RCSQ variables during three days of ICU stay. Data are expressed as mean ± SD. ^#^: The analysis of variance test was applied for repeated measures. A p-value <0.05 is considered significant. RCSQ: Richards-Campbell Sleep Questionnaire; ICU: intensive care unit

RCSQ variables	Day 1	Day 2	Day 3	Test statistics	P-value
Sleep depth	51.37 ± 8.67	48.37 ± 9.06	48.5 ± 8.87	f = 2.90	0.057^#^
Sleep latency	50.62 ± 11.62	47.62 ± 9.97	49.25 ± 7.25	f = 1.95	0.146^#^
Awakening	49.5 ± 10.17	48.25 ± 9.52	50.62 ± 8.01	f = 1.35	0.262^#^
Returning to sleep	49 ± 10.14	47.5 ± 10.12	49.5 ± 10.42	f = 0.84	0.432^#^
Sleep quality	47.5 ± 10.12	49.62 ± 9.06	50.75 ± 11.22	f = 2.2	0.118^#^
Noise	51.25 ± 11.07	51.75 ± 9.51	51.37 ± 8.68	f = 0.7	0.932^#^
Mean RCSQ	49.87 ± 5.35	48.85 ± 4.79	50 ± 4.67	f = 1.75	0.177^#^

Sleep depth scores were 51.37 ± 8.67 on day one, 48.37 ± 9.06 on day two, and 48.5 ± 8.87 on day three (p = 0.057). Sleep latency scores showed a slight decline from 50.62 ± 11.62 on day one to 47.62 ± 9.97 on day two, followed by mild improvement on day three (49.25 ± 7.25), though this was not statistically significant (p = 0.146). Similarly, awakening frequency and the ability to return to sleep remained largely unchanged over the study period. Perceived sleep quality improved marginally from 47.5 ± 10.12 on day one to 50.75 ± 11.22 on day three; however, this difference was not statistically significant (p = 0.118). Noise perception scores remained almost constant across the three days (p = 0.932), suggesting persistent environmental disturbance in the ICU setting.

Noise measurement was consistently elevated within the ICU. Morning noise levels ranged from 54.59 ± 6.74 dB on day one to 53.3 ± 4.12 dB on day three, while evening noise levels ranged between 42.54 ± 1.82 dB and 44.38 ± 3.4 dB. No statistically significant changes in noise levels were observed over the three days (p = 0.155 for morning and p = 0.521 for evening measurements). Pain scores assessed using the NPRS demonstrated a statistically significant reduction over time. The mean pain score decreased from 5.87 ± 0.9 on day one to 5.22 ± 0.75 on day three (p = 0.001) (Table 4).

**Table 4:** Noise levels and pain scores over the three days in the ICU. Data expressed as mean ± SD^.^ ^#^: Analysis of variance test was applied for repeated measures. A p-value <0.05 was considered significant. NPRS: Numeric Pain Rating Scale; ICU: intensive care unit

Parameter		Day 1	Day 2	Day 3	Test statistics	P-value
Noise level (dB)	Morning	54.59 ± 6.74	53.37 ± 4.09	53.3 ± 4.12	f = 1.83	0.155^#^
	Evening	44.02 ± 3.57	44.38 ± 3.4	42.54 ± 1.82	f = 0.84	0.521^#^
Pain score (NPRS)		5.87 ± 0.9	5.7 ± 1.02	5.22 ± 0.75	f = 17.39	0.001^#^

Delirium was identified in 16 (8.0%) patients on day one, 14 (7.6%) patients on day two, and 10 (5.9%) patients on day three, demonstrating a gradual decline in incidence over the study period. Overall, the cumulative incidence of delirium during the three-day observation period was 20%.

## Discussion

This observational study evaluated sleep quality among trauma patients admitted to the ICU using the RCSQ. The RCSQ has been validated in numerous studies as a reliable instrument for assessing sleep quality in critically ill patients [6-9]. However, its main limitation is that it offers only a subjective assessment of sleep and does not objectively measure sleep patterns or physiological sleep parameters. This study demonstrated that sleep disturbance is highly prevalent among trauma ICU patients, with more than half of the patients experiencing poor sleep quality during the first three days of ICU admission. The mean RCSQ score on day one in our cohort was 49.87 ± 5.35 (and over three days was 49.57 ± 4.95), indicating overall poor sleep quality. These findings have a considerable effect as sleep plays an important role in physical restoration, immune modulation, neurocognitive function, hormonal regulation, and recovery from critical illness. Evidence indicates that sleep deprivation has an adverse impact on metabolic clearance from the brain, inflammatory pathways modulation, and cardiovascular function [1,2,14].

Similar findings have been reported in multiple ICU studies. Naik et al. observed a mean RCSQ score of 51.6 ± 13.5 among ICU patients and classified patients with scores below 50 as poor sleepers [25]. Carrera-Hernández et al. also demonstrated that critically ill patients frequently perceived their ICU sleep to be poor because of repeated awakenings and environmental disturbances [26]. Likewise, Özkan et al. found poor sleep quality to be highly prevalent among surgical ICU patients [27]. The mean RCSQ scores observed in our trauma cohort were slightly lower than those reported in general ICU populations, indicating poor sleep. This difference may be attributed to trauma-specific factors such as pain, fractures, multiple injuries, and emotional factors. Trauma patients commonly experience increased sympathetic activity and an inflammatory response that impairs the sleep cycle. Furthermore, orthopedic immobilization, thoracic trauma, the presence of drains, and postoperative pain may substantially impede both the onset and continuity of sleep.

In the present study, poor sleep quality (RCSQ <50) was observed in 52.5% (n = 42) of patients on day one, 45% (n = 36) on day two, and 43.75% (n = 35) on day three. Although there was a marginal reduction in the proportion of poor sleepers over time, overall sleep quality remained persistently impaired throughout ICU stay. However, this modest improvement over the initial three days suggests that sleep quality may gradually enhance as patients stabilize and adapt to the ICU environment, although it remained poor overall.

The use of the RCSQ in the present study provided a practical and feasible method for sleep assessment in critically ill trauma patients. Polysomnography remains the gold standard for sleep evaluation; however, its application in ICU settings is technically difficult and resource-intensive [4]. Several studies have validated the utility of RCSQ in ICU populations [5-9]. Richards et al. originally demonstrated good reliability and validity of the RCSQ when compared with polysomnography [5]. Kamdar et al. also reported acceptable interrater agreement between patients and nursing staff using the questionnaire [8]. More recent studies by Locihová et al. [28] and Lkoul et al. [29] further confirmed the utility of RCSQ in ICU sleep assessment compared with actigraphy and polysomnography.

The present study found no significant association between sleep quality and demographic variables such as age and gender. Similarly, illness severity scores such as APACHE II, SOFA, and ISS did not significantly differ between good and poor sleep groups. These findings suggest that sleep quality impairment in ICU patients may occur irrespective of baseline illness severity. Likewise, Ahn et al. reported that ICU sleep quality is influenced more by environmental and treatment-related factors rather than severity scores alone [30]. Lewandowska et al. also observed that environmental conditions and ICU care practices were stronger determinants of sleep disturbance than demographic characteristics [31].

Factors affecting sleep

In our study, noise (65%) was the most important factor disturbing sleep, followed by pain (46.25%) and light (22.5%). Other less common factors included discomfort caused by catheters, monitor connections, intravenous lines, and frequent nursing interventions such as medication and fluid administration. Multiple studies have emphasized the multifactorial causes of sleep disruption in the ICU, but different authors have studied different factors. Carrera-Hernandez et al., in an observational study in ICU patients, reported pain as the most important factor affecting quality of sleep (p = 0.009) [26].

A few other study findings partially align with, yet differ from, previous reports in the literature. Ahn et al. [30] also reported physical discomfort and awakening due to medical procedures (43% each), followed by feeling unwell from the underlying diseases (37%). Other factors, such as noise (30%) and light (13%), were less significant than patient-related factors [30]. Similarly, Lewandowska et al. identified frequent measurement of vital signs as the most common sleep disruptor in the ICU, followed by light, blood sampling, pain, diagnostic procedures, and noise [31]. Interestingly, noise ranked only last in their observations. Bano et al. demonstrated that environmental factors such as noise, lighting, and interruptions contribute significantly to poor sleep in hospitalized patients [16]. However, Longley et al. observed that sleep disturbance in surgical trauma ICU patients remained substantial despite pain management, highlighting the importance of non-pain-associated factors [32]. Therefore, sleep quality in trauma ICU patients requires a multidimensional approach rather than analgesic optimization alone.

Noise and sleep quality

Environmental noise is one of the most extensively studied factors of ICU sleep disruption. In our study, ICU noise levels consistently exceeded the World Health Organization (WHO) recommendations [33]. Morning noise levels remained above 53 dB across all three days, whereas recommended morning-time hospital noise levels are below 35-40 dB. Evening noise levels also remained elevated. Continuous noise exposure interferes with the sleep cycle, causes frequent awakenings, and disrupts REM sleep.

Diurnal variations of noise level were noted in the ICU, indicating significant noise level during the day compared to the night (54.59 ± 6.74 dB vs. 44.02 ± 3.57 dB). Eliott et al. observed distinct diurnal variations, reporting an average noise level of 53.95 dB during the day compared to 50.20 dB at night, with nighttime levels significantly higher than those recorded in our ICU during the same period [9]. This increased noise during the day can be attributed to multiple factors, such as greater staff activity, medical rounds, various intervention procedures, physiotherapist visits, and the presence of patient attendants. No previous studies were identified that specifically compared morning versus evening noise levels in the ICU.

Our findings are consistent with prior literature evaluating ICU noise and sleep quality. Gabor et al. demonstrated that ICU environmental noise significantly contributes to sleep fragmentation in mechanically ventilated patients [15]. Gulam et al. similarly reported persistent excessive noise levels in trauma and orthopedic wards, adversely affecting sleep quality [34]. Jaiswal et al. found ICU sound levels to be consistently disruptive and comparable to non-ICU hospital wards [35]. Furthermore, Scquizzato et al. validated the feasibility of using Apple-based devices for ICU noise assessment, supporting the methodology employed in our study [24].

Interestingly, despite persistent noise exposure, noise perception scores on the RCSQ remained relatively stable over the three days. This may suggest acclimatization to ICU surroundings over time, although objective sleep quality remained impaired. Continuous alarm sounds, staff communication, equipment movement, and nighttime patient care activities likely contributed to sustained sleep fragmentation throughout ICU stay.

Delirium in trauma intensive care units

Sleep disturbance and delirium are closely interrelated in critically ill patients. Poor sleep patients are more predisposed to delirium through disruption in circadian rhythm, imbalance in neurotransmitters, and neurological inflammation [36]. Trauma patients are prone to delirium because of pain, sedation exposure, multiple surgeries, and inflammatory response. Marquetand et al. reported a significant incidence of delirium (21.7%) among trauma patients and emphasized the importance of identifying modifiable risk factors [37]. In our study, delirious patients were excluded during enrolment to minimize confounding and allow better evaluation of sleep quality independently. However, the incidence of delirium observed in our study was lower. This rate is lower than that reported in other studies. In contrast, van der Hoeven et al. [38] in a retrospective analysis of 323 ICU patients reported an incidence of 45.8%, and a meta-analysis involving 39,076 ICU patients reported a delirium incidence of 33% [36]. The lower incidence of delirium in our study may be partially attributed to a relatively younger patient population, the predominant use of fentanyl as sedation, a smaller proportion of mechanically ventilated patients (n = 24; 30%), and the mean RCSQ score slightly below 50.

Mechanical ventilation is another important factor implicated in ICU sleep disturbance. Ventilator dyssynchrony, sedation practices, airway discomfort, and alarms may adversely affect sleep architecture [9]. However, in our study, mechanical ventilation was not significantly associated with poor sleep quality. This may be due to the relatively small proportion of ventilated patients, exclusion of deeply sedated patients, and modern ICU sedation strategies that may have minimized sleep disruption. Elliott et al. demonstrated that ICU patients often experience abnormal sleep architecture irrespective of ventilatory status, suggesting that multiple ICU-related factors contribute simultaneously to sleep impairment [9]. Another important finding in the present study was the higher mortality among poor sleepers when compared with good sleepers (16.67% vs. 5.26%); however, the difference was not statistically significant. Previous literature has shown that sleep deprivation may result in adverse ICU outcomes through immunological dysfunction, increased delirium risk, delayed healing, and neurocognitive disturbances [13,14]. Recently, van der Hoeven et al. [38] demonstrated associations between ICU sleep disturbance, delirium, and mortality. Therefore, the trend toward higher mortality among poor sleepers observed in our study may still hold clinical significance despite statistical non-significance.

Strengths and limitations

The present study has several strengths. It specifically focused on trauma ICU patients, a cohort in whom sleep quality has not been extensively studied. The prospective design, repeated sleep assessment over three days, simultaneous measurement of pain and environmental noise, and use of validated sleep assessment tools strengthen the reliability of the findings. Furthermore, ICU noise measurement using the NIOSH sound level meter application provided an objective noise assessment. There are certain limitations to acknowledge in this study. First, the relatively small sample size reduces the applicability of our results to the wider ICU population and to detecting sleep quality and mortality. Second, sleep assessment was subjective and based on RCSQ rather than polysomnography, which is cumbersome to use. Although RCSQ is validated and clinically practical, it cannot provide detailed information regarding sleep patterns or REM sleep alterations. Third, the study was conducted in a single tertiary trauma center, limiting its generalizability. Fourth, a short three-day follow-up sleep study period restricted insights into the long-term effects of sleep quality on recovery and mortality. Finally, factors such as sedative use, nursing interventions, nighttime procedures, anxiety, and psychological stress were not examined quantitatively.

## Conclusions

This study demonstrated that trauma patients admitted to the ICU experience poor sleep quality, as assessed by the RCSQ. Further, sleep quality showed a gradual improvement over the course of their ICU stay, but it remained poor. Noise levels in the ICU were consistently above the recommended permissible limits, with significantly higher noise levels reported during the daytime compared with nighttime. Among the factors affecting sleep, noise was the most common, followed by light and pain related to traumatic injuries. These findings suggest that better management of sleep is required in trauma patients to improve the clinical outcome. Although sleep quality did not significantly influence mortality in this study, larger multicenter trials are required to substantiate this finding and further explore the relationship between sleep quality and clinical outcomes in trauma ICU patients.

## References

[1] Helton MC, Gordon SH, Nunnery SL (1980). The correlation between sleep deprivation and the intensive care unit syndrome. Heart Lung.

[2] Xie L, Kang H, Xu Q (2013). Sleep drives metabolite clearance from the adult brain. Science.

[3] Collop NA, Salas RE, Delayo M, Gamaldo C (2008). Normal sleep and circadian processes. Crit Care Clin.

[4] Knauert MP, Yaggi HK, Redeker NS, Murphy TE, Araujo KL, Pisani MA (2014). Feasibility study of unattended polysomnography in medical intensive care unit patients. Heart Lung.

[5] Richards KC, O'Sullivan PS, Phillips RL (2000). Measurement of sleep in critically ill patients. J Nurs Meas.

[6] Darbyshire JL, Borthwick M, Edmonds P, Vollam S, Hinton L, Young JD (2020). Measuring sleep in the intensive care unit: electroencephalogram, actigraphy, or questionnaire?. J Intensive Care Soc.

[7] Menear A, Elliott R, M Aitken L, Lal S, McKinley S (2017). Repeated sleep-quality assessment and use of sleep-promoting interventions in ICU. Nurs Crit Care.

[8] Kamdar BB, Shah PA, King LM (2012). Patient-nurse interrater reliability and agreement of the Richards-Campbell sleep questionnaire. Am J Crit Care.

[9] Elliott R, McKinley S, Cistulli P, Fien M (2013). Characterisation of sleep in intensive care using 24-hour polysomnography: an observational study. Crit Care.

[10] Biazim SK, Souza DA, Carraro Junior H, Richards K, Valderramas S (2020). The Richards-Campbell Sleep Questionnaire and Sleep in the Intensive Care Unit Questionnaire: translation to Portuguese and cross-cultural adaptation for use in Brazil. J Bras Pneumol.

[11] Parthasarathy S, Tobin MJ (2004). Sleep in the intensive care unit. Intensive Care Med.

[12] Pisani MA, Friese RS, Gehlbach BK, Schwab RJ, Weinhouse GL, Jones SF (2015). Sleep in the intensive care unit. Am J Respir Crit Care Med.

[13] Altman MT, Knauert MP, Pisani MA (2017). Sleep disturbance after hospitalization and critical illness: a systematic review. Ann Am Thorac Soc.

[14] Grandner MA, Sands-Lincoln MR, Pak VM, Garland SN (2013). Sleep duration, cardiovascular disease, and proinflammatory biomarkers. Nat Sci Sleep.

[15] Gabor JY, Cooper AB, Crombach SA, Lee B, Kadikar N, Bettger HE, Hanly PJ (2003). Contribution of the intensive care unit environment to sleep disruption in mechanically ventilated patients and healthy subjects. Am J Respir Crit Care Med.

[16] Bano M, Chiaromanni F, Corrias M (2014). The influence of environmental factors on sleep quality in hospitalized medical patients. Front Neurol.

[17] Sessler CN, Gosnell MS, Grap MJ (2002). The Richmond Agitation-Sedation Scale: validity and reliability in adult intensive care unit patients. Am J Respir Crit Care Med.

[18] Ely EW, Inouye SK, Bernard GR (2001). Delirium in mechanically ventilated patients: validity and reliability of the confusion assessment method for the intensive care unit (CAM-ICU). JAMA.

[19] Breivik H, Borchgrevink PC, Allen SM (2008). Assessment of pain. Br J Anaesth.

[20] Knaus WA, Draper EA, Wagner DP, Zimmerman JE (1985). APACHE II: a severity of disease classification system. Crit Care Med.

[21] Vincent JL, Moreno R, Takala J (1996). The SOFA (Sepsis-related Organ Failure Assessment) score to describe organ dysfunction/failure. On behalf of the Working Group on Sepsis-Related Problems of the European Society of Intensive Care Medicine. Intensive Care Med.

[22] VanDerHeyden N, Cox TB (2008). Trauma scoring. Current Therapy of Trauma and Surgical Critical Care.

[23] Crossley E, Biggs T, Brown P, Singh T (2021). The accuracy of iPhone applications to monitor environmental noise levels. Laryngoscope.

[24] Scquizzato T, Gazzato A, Landoni G, Zangrillo A (2020). Assessment of noise levels in the intensive care unit using Apple Watch. Crit Care.

[25] Naik RD, Gupta K, Soneja M, Elavarasi A, Sreenivas V, Sinha S (2018). Sleep quality and quantity in intensive care unit patients: a cross-sectional study. Indian J Crit Care Med.

[26] Carrera-Hernández L, Aizpitarte-Pejenaute E, Zugazagoitia-Ciarrusta N, Goñi-Viguria R (2018). Patients' perceptions of sleep in a critical care unit. Enferm Intensiva (Engl Ed).

[27] Özkan ZK, Dığın F, Kalaycı E (2023). Sleep quality and related factors in surgical intensive care patients. Türk Uyku Tıbbı Dergisi.

[28] Locihová H, Axmann K, Žiaková K, Šerková D, Černochová S (2020). Sleep quality assessment in intensive care: actigraphy vs. Richards-Campbell sleep questionnaire. Sleep Sci.

[29] Lkoul A, Oumbarek K, Bouchriti Y, Jniene A, Dendane T (2025). Sleep quality assessment in intensive care units: comparing actigraphy and the Richards Campbell Sleep Questionnaire-a pilot study in the Moroccan context. Clocks Sleep.

[30] Ahn YH, Lee HY, Lee SM, Lee J (2023). Factors influencing sleep quality in the intensive care unit: a descriptive pilot study in Korea. Acute Crit Care.

[31] Lewandowska K, Mędrzycka-Dąbrowska W, Kwiecień-Jaguś K (2019). Factors determining sleep in patients hospitalised in ICUs in a hospital in Northern Poland. Sleep Biol Rhythms.

[32] Longley L, Simons T, Glanzer L, Du C, Trinks H, Letzkus L, Quatrara B (2018). Evaluating sleep in a surgical trauma burn intensive care unit: an elusive dilemma. Dimens Crit Care Nurs.

[33] Schwela DH (2001). The new World Health Organization guidelines for community noise. Noise Control Eng J.

[34] Gulam S, Xyrichis A, Lee GA (2020). Still too noisy - an audit of sleep quality in trauma and orthopaedic patients. Int Emerg Nurs.

[35] Jaiswal SJ, Garcia S, Owens RL (2017). Sound and light levels are similarly disruptive in ICU and non-ICU wards. J Hosp Med.

[36] Wu NN, Zhang YB, Wang SY, Zhao YH, Zhong XM (2023). Incidence, prevalence and risk factors of delirium in ICU patients: a systematic review and meta-analysis. Nurs Crit Care.

[37] Marquetand J, Gehrke S, Bode L (2022). Delirium in trauma patients: a 1-year prospective cohort study of 2026 patients. Eur J Trauma Emerg Surg.

[38] van der Hoeven AE, Bijlenga D, van der Hoeven E (2024). Sleep in the intensive and intermediate care units: exploring related factors of delirium, benzodiazepine use and mortality. Intensive Crit Care Nurs.

